# High *In-Vitro* Antitumour Activity of Triphenyltin Coumarin 3-Carboxylate and its Coordination Complexes With Monodentate Oxygen Donor Ligands Against the Epstein Barr Virus (EBV)-DNA Positive Raji and the P-388 Murine Leukaemia Cell Lines, and Evidence for the Suppression by Organotin of the Early Antigen Complex in the EBV Lytic Cycle

**DOI:** 10.1155/MBD.2000.245

**Published:** 2000

**Authors:** Joyce Koshy, V. G. Kumar Das, S. Balabaskaran, S. W. Ng, Norhanom Wahab

**Affiliations:** 1 Institute for Postgraduate Studies and Research University of Malaya Kuala Lumpur 50603 Malaysia; 2 Department of Chemistry University of Malaya Kuala Lumpur 50603 Malaysia; 3 Department of Biochemistry University of Malaya Kuala Lumpur 50603 Malaysia; 4 Institute of Biological Sciences University of Malaya Kuala Lumpur 50603 Malaysia

## Abstract

Triphenyltin coumarin-3-carboxylate and its coordination complexes with ethanol, triphenylphosphine oxide, triphenylarsine oxide, diphenylcyclopropenone and quinoline N-oxide exhibited high *in vitro* cytotoxicity (LC_50_ values in the range 0.25-3.4 μg/mL) when tested against EBV-DNA positive Raji cells and P-388 leukaemia cells, compared to the standard drug 5-Fluorouracil, which showed LC_50_ values of 11 and >50 μg/mL, respectively, against these cells. Additional tests performed on the Raji cells incubated with the quinoline *N*-oxide complex in the presence of the tumour promoters, TPA and sodium butyrate, revealed that the *diffused* and *restricted* protein components of the early antigen complex were suppressed relative to the control containing only the promoters, indicating impaired function of the genes involved as transactivators in the early lytic cycle of the EBV. The failure of the restriction enzymes *Eco R1* and *Hind III* to cleave the extracted DNA from such treated cells in contrast to the control, coupled with the amplification of the BMLF-1 gene by the PCR technique which was realised only with the DNA of the control and not of the treated sample, point to a punitive interaction of the organotin with the nuclear DNA of the Raji cells.


### HIGH *IN-VITRO* ANTITUMOUR ACTIVITY OF TRIPHENYLTIN COUMARIN - 3-CARBOXYLATE AND ITS COORDINATION COMPLEXES WITH MONODENTATE OXYGEN DONOR LIGANDS AGAINST THE EPSTEIN BARR VIRUS (EBV)- DNA POSITIVE RAJI AND THE P-388 MURINE LEUKAEMIA CELL LINES, AND EVIDENCE FOR THE SUPPRESSION BY ORGANOTIN OF THE EARLY ANTIGEN COMPLEX IN THE EBV LYTIC CYCLE

Joyce Koshy,[1] V. G. Kumar Das*,[2] S. Balabaskaran,[3] S. W. Ng[1] and Norhanom Wahab[4]

[1] Institute for Postgraduate Studies and Research, [2] Department of Chemistry, [3] Department of Biochemistry, [4] Institute of Biological Sciences, University of Malaya, 50603 Kuala Lumpur, Malaysia

**Abstract**
Triphenyltin coumarin-3-carboxylate and its coordination complexes with ethanol, triphenylphosphine oxide, triphenylarsine oxide, diphenylcyclopropenone and quinoline *N*-oxide exhibited high *in vitro* cytotoxicity ($LC_{50}$ values in the range 0.25-3.4 µg/mL) when tested against EBV-DNA positive Raji cells and P-388 leukaemia cells, compared to the standard drug 5-Fluorouracil, which showed $LC_{50}$ values of 11 and >50 µg/mL, respectively, against these cells. Additional tests performed on the Raji cells incubated with the quinoline *N*-oxide complex in the presence of the tumour promoters, TPA and sodium butyrate, revealed that the *diffused* and *restricted* protein components of the early antigen complex were suppressed relative to the control containing only the promoters, indicating impaired function of the genes involved as transactivators in the early lytic cycle of the EBV. The failure of the restriction enzymes *Eco R1* and *Hind III* to cleave the extracted DNA from such treated cells in contrast to the control, coupled with the amplification of the BMLF-1 gene by the PCR technique which was realised only with the DNA of the control and not of the treated sample, point to a punitive interaction of the organotin with the nuclear DNA of the Raji cells.

### Introduction

A large number of di-and tri-organotin compounds exhibit *in vitro* anti-tumour properties against a wide variety of human tumour cell lines[1-6], including Ehrlich ascites tumour, IMC carcinoma, P-388 lymphocytic leukaemia, Sarcoma 180, MCF-7 and EVSA-T (mammary tumours), WiDr (a colon cancer), IGROV (an ovarian cancer), A 498 (a renal cancer), M19 MEL (a melanoma) and H 226 (a lung cancer). However, there is no previous study on nasopharyngeal carcinoma (NPC) involving organotins. This disease has much epidemiological significance in Southeast Asia and the Far East on account of the genetic predisposition of the malignancy among the Chinese population[7].

Malignant neoplasms in the nasopharynx is a heterogeneous group of tumours, among which are certain types which are associated with the Epstein Barr virus (EBV)[8-9], a B lymphotropic human herpes virus which is transmitted primarily in saliva or, less commonly, by blood transfusion. The virus is often found in the latent state in many human cell lines, but can be induced into the lytic cycle by a variety of agents[10], including superinfection with the P3HR-1 strain of EBV, phorbol ester (12,*O*-tetradecanoylphorbol −13-acetate [TPA]) or sodium butyrate. The mechanism of the induction by TPA is not well understood, but there is evidence that the induction is at the transcriptional level[11]. A number of genes in the EBV genome, among them BZLF-1, BMLF-1, BMRF-1 and BRLF-1 have been identified as transactivators in the early lytic cycle[10, 12]. Of these, the BZLF-1 and BMLF-1 genes are considered to play major control roles in the switch from the latent to the lytic viral cycle[11]. The BMLF-1 gene, which encodes one of the early antigen (EA) proteins that is expressed in the lytic state, has been shown to have a promoter region and two well defined activation sites upstream to the transcription start site that are inducible by the phorbol ester[11].

In this study, we have investigated the *in vitro* cytotoxic effects of triphenyltin coumarin 3-carboxylate and its coordination complexes with monodentate oxygen donor ligands against the EBV-DNA positive Raji and the P-388 murine leukaemia cancer cell lines. The common chemotherapeutic drug, 5-Fluorouracil, was included in the screening tests. [Triphenyltin coumarin -3-carboxylate •Quinoline-*N* –oxide], which proved to be the most active against the Raji cells, was selected for a detailed investigation of its effect on the expression of the EBV-EA complex in the lytic cycle of the virus induced by the combined use[13] of TPA and sodium *n*-butyrate. Using anti-EA polyclonal antibodies derived from patients with NPC as probes, we have demonstrated that the organotin suppressed both the *diffused* and *restricted* components[14] of the EA complex. In the context of the inhibition of TPA induction, we have also examined the integrity of the DNA isolated from the treated Raji cells relative to the control, including that of the PCR-amplified BMLF-1 gene fragment in both situations. Our observations here are included in this paper.

### Materials and Methods

***Materials***

Triphenyltin coumarin-3-carboxylate and its 1:1 or 2:1 monomeric complexes obtained when the Lewis acid is mixed with equimolar amounts of various monodentate oxygen donor ligands were synthesised as previously reported[15]. TPA, sodium *n*-butyrate and 5-Fluorouracil were Sigma products. The NPC cell line used was the EBV-DNA positive Raji cell line obtained from NCI (Bethesda); the P-388 cell line was procured from American Type Culture Collection. The cells were routinely maintained as per standard protocol in RPMI 1640 and Dulbecco minimum essential medium, respectively, with each medium supplemented with 10% fetal calf serum and antibiotics.

***Cytotoxicity assay***

Cells growing in the logarithmic phase were used in the assay. Each well of a 96- well microtitre plate was charged with 300 µL of the cell suspension containing approximately $2x10^5$ cells. The cells, with the exception of the control, were incubated with 3 µL of the test solutions in the concentration range 500 ng/mL to 10 µg/mL made up in DMSO. The plate was placed in a humidified incubator (37 °C; 5% $CO_2$ ) for 24-48 h. A 1:1 ratio of cell suspension to Tryphan Blue (0.1% solution in phosphate buffered saline [PBS] ) was then prepared in a separate microtitre plate, and a drop of this was placed on a haemocytometer for viewing and cell count using an inverted microscope (20x objective). Only dead cells took up the blue stain. Plots of percentage inhibition (based on the ratio, Number of cells alive /Total cell count) against the concentrations of the test compound used yielded $LC_{50}$ (concentration at which the test compounds caused one-half of the cells to be killed) values. An average of 2-3 readings for each concentration of the test compound was used for the cytotoxicity evaluation.

***Indirect Immunofluorescence Assay***

Raji cells in the logarithmic phase and at a density of $2x10^5$ cells/mL were incubated with the organotin test compound at various concentrations between the $ID_{50}$ value and 10 µg/mL in the presence of the promoters 20 ng/mL TPA and 4 mM/mL sodium butyrate. The control contained only the promoters. The cells were incubated for 72 h in a humidified incubator (37 °C; 5% $CO_2$) before being harvested. A drop of the cell suspension (in 100 µL PBS) was placed into each well of a multitest Teflon-coated slide and fixed with acetone. 20 µL of EBV-EA positive serum obtained from patients suffering from nasopharyngeal carcinoma (polyclonal antibodies), previously diluted 20 fold in PBS, was dispensed into each well and the bound antibody detected using a second antibody against the first, in this case, goat anti-human IgG, previously labelled with fluorescein- isothiocyanate. The fluorescent Raji cells embodying antigen-antibody interactions were observed under an UV microscope; their number decreased markedly when higher concentrations of organotin were used in the treatment.

***Detection of Early Antigen protein suppression by the Western Blot technique***

Control and organotin-treated Raji cells were washed with PBS at 4 °C and, in the presence of an equal volume of the detergent sodium dodecyl sulphate (SDS), heated at 100 °C for 10 min. After cooling, the chromosomal DNA was sheared by sonication. The sample was then centrifuged at 10,000g for 10 min. and the supernatant containing the cellular protein collected. Approximately 100 µg of the protein was loaded onto a 0.75 mm thick 12% SDS polyacrylamide gel and electrophoresis performed on it using 100 V. Prestained SDS-PAGE standards of low molecular weight were used as protein markers. The separated protein bands on the gel were then transferred (blotted) on to a 0.2 µ thick Immobilon polyvinylidene difluoride (PVDF) membrane electrophoretically by sandwiching the gel and the membrane, which had previously been soaked in a transfer buffer, between two electrodes. Transfer was achieved in approximately 1h using 21 V. The membrane was removed and soaked for 1-2 h in a blocking solution of non-fat milk in PBS to block any subsequent, non-specific adsorption of antibodies by the membrane. The membrane was then incubated overnight with NPC serum previously diluted 200 fold using the blocking solution. Excess antibodies were then washed from the membrane and the bound antibody which remained was detected using a second antibody, namely affinity- purified goat anti-human IgG horseradish peroxidase conjugate (BIO-RAD), diluted in phosphate-free blocking solution. Following incubation for 1 h, the membrane was soaked for 10 min in a solution of 150 mM NaCl and 50 mM Tris-HCl and then immersed in the HRP colour development solution. After the bands appeared, the membrane was air dried and photographed.

***DNA extraction and analysis***

Control and organotin-treated Raji cells were washed with PBS and centrifuged as described in the previous section. The supernatant was discarded and the residue was treated with 500 µL of the lysis buffer. Standard laboratory procedures[16] were followed in removing protein and RNA by sequential incubations with proteinase K and ribonuclease A, and in extracting the DNA finally into ethanol. The extracted DNA was analysed by electrophoresis using 0.8% agarose gel. The location of the DNA bands within the gel was facilitated by including 0.5 µg/mL of the fluorescent dye ethidium bromide in the gel. 1 kb DNA ladder and λ *Hind III* markers were used as reference.

***Digestion of Plasmid pBR322 with (a) organotin and (b) Hind III***

PBR 322 bacterial plasmid was incubated with the restriction enzyme *Hind III* digest as well as separately with various concentrations of [triphenyltin coumarin-3-acetate •Quinoline *N*-oxide] in Eppendorf tubes at 37 °C for 2 h in a water bath. At the end of the incubation period, the samples were subjected to electrophoresis using 1.4% agarose gel containing a small amount of ethidium bromide, and the fluorescent DNA bands observed under UV light.

***Digestion of Raji DNA with Eco RI and Hind III restriction enzymes***

Extracted DNA (6.0 μL) from Raji cells, both organotin-treated and control, were incubated with *Eco R1* and *Hind III* (each 3.0 μL) in separate experiments in the presence of the standard restriction enzyme digestion mixture, and at a temperature of 37 °C for 2 h. The samples were then subjected to electrophoresis as described above.

***Amplification of the BMLF-1 gene by Polymerase Chain Reaction (PCR)***

Template DNA (5.0 μL) from control and organotin-treated Raji cells was mixed with EBV BMLF-1 primer set, deoxynucleotide triphosphate and Taq DNA polymerase (reagents obtained from Maxim Biotech, Inc.), and the PCR carried out for 35 cycles as per standard protocol[17]. Each cycle of PCR consisted of thermal denaturation at 94 °C, primer annealing at 55 °C and extension at 72 °C, all carried out in the automated thermal cycler (Perkin- Elmer Cetus). The amplified samples were then purified just once employing the PURE-GENE PCR purification kit and then subjected to electrophoresis on 1.5% agarose gel containing 0.5 μg/mL ethidium bromide. DNA ladders of 100 bp as well as 1 kbp were used as markers to detect the characteristic 265 bp band of the BMLF-1 gene.

**Results and Discussion**

*Cytotoxicity assay*

The $LC_{50}$ values of the organotin compounds screened against the Raji and P-388 cell lines are shown in Table 1. The data clearly show that against both cancer cell lines the organotins (all pentacoordinate at tin) were significantly more cytotoxic than the standard drug , 5-Fluorouracil. The organotins appeared to be all uniformly active against the Raji cells, with the quinoline- *N*-oxide complex, [TPTCC•QuinO],

**Table 1. Cytotoxicity data for triphenyltin coumarin-3-carboxylate and its coordination complexes with monodentate ligands against the Raji and P-388 cell lines in comparison with 5-Fluorouracil**

| Compounds | Raji cells ($LC_{50}$ μg/mL) | P-388 cells ($LC_{50}$ μg/mL) |
|---|---|---|
| Triphenyltin coumarin-3-carboxylate (TPTCC) | 1.05 | 3.4 |
| [TPTCC][TPTCC• $Ph_3PO$] | 1.0 | 0.75 |
| [TPTCC• EtOH] | 1.2 | 2.1 |
| [TPTCC][TPTCC • $Ph_3AsO$] | 0.65 | 0.25 |
| [TPTCC• DPCP] | 1.0 | 0.35 |
| [TPTCC• QuinO] | 0.45 | 1.75 |
| 5-Fluorouracil | >50 | 11.0 |

registering marginally greater activity than the others. This compound, therefore, was chosen for further experiments with the Raji cells. Against the P-388 cell line, the organotin complexes were more active than the parent organotin carboxylate Lewis acid, with the complexes containing the ligands triphenylarsine oxide and diphenyl cyclopropenone (DPCP) exhibiting greater activities. Whereas a concentration of 11μg/mL of 5-Fluorouracil was required to obtain 50% inhibition of growth of the P-388 cells, approximately the same concentration of [TPTCC•QuinO] caused 100% inhibition.

***Inhibition of EBV-Early Antigen proteins by organotin***

Incubation of the EBV genome-positive Raji cells in the logarithmic growth phase with TPA and sodium *n*-butyrate caused the induction of the lytic viral cycle with expression of the early antigen complex (EA). This was evidenced by the successful binding of the EA to the added polyclonal antibodies introduced from the EBV-EA positive serum obtained from patients suffering from nasopharyngeal carcinoma, as detected by the indirect immunofluorescence assay. Relative to the control (*Fig.1, left*), the number of fluorescent Raji cells embodying antigen-antibody interactions decreased in the organotin-treated case, with the inhibitory effect being more pronounced when higher organotin concentrations were used in the treatment. Concentrations of 50,100,200 and 400 ng/mL of the organotin, [TPTCC•QuinO], were used in the study; total suppression of the EBV-EA was achieved at the 200 ng/mL concentration level (*Fig.1, right*). With the triphenylarsine oxide complex, completely non-fluorescent cells were obtained only at the treatment concentration of 600 ng/mL.

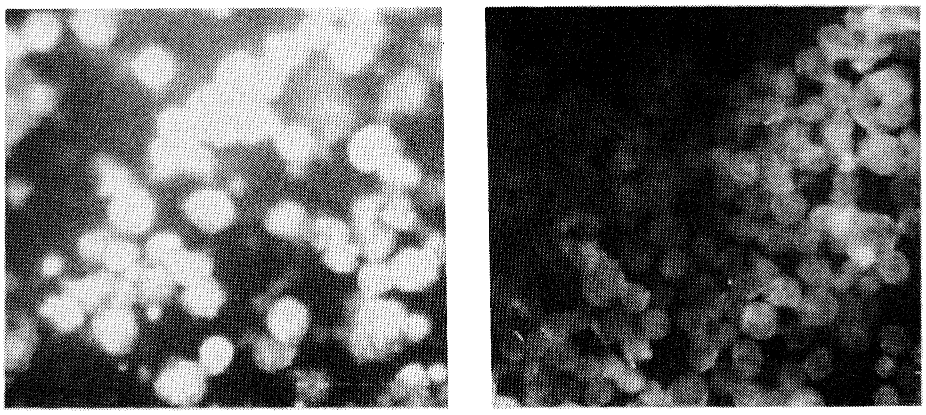

**Fig. 1: Immunofluorescence staining patterns in (*left*) untreated Raji cells (control) showing induction of EBV-EA by TPA and sodium *n*-butyrate and (*right*) Raji cells treated with [TPTCC•QuinO] at 200 ng/ml concentration showing absence of EBV-EA induction.**

The Western Blot technique was next used to investigate whether the organotin affected either or both the *restricted* and *diffused* protein components of the early antigen complex. Previous studies using monoclonal antibodies have conclusively demonstrated[14,18] that the 85 kDa protein is a distinguishing characteristic of EA(R), a methanol-sensitive antigen restricted to the cytoplasm, while two major protein bands in molecular weight range 47-56 kDa are associated with EA(D), a methanol resistant and largely intranuclear antigen. *Fig. 2* depicts the Western Blot in which lanes 6-9 relate to protein bands obtained from Raji cells treated with [TPTCC•QuinO]. The suppression of both EA(R) and EA(D) is evident at the higher organotin concentrations of 200 and 400 ng/mL.

Conceivably, the concentration-dependent inhibitory effect of the organotin could arise as a result of the blockage of the TPA-and-sodium butyrate promotion of the genes that function as transactivators in the early lytic cycle of the virus. As pointed out in the Introduction, one of these is the BMLF-1 gene. This implies interaction of the organotin with the nuclear DNA and hence with the resident EBV genome to varying levels during incubation. To test this point, we examined the integrity of the DNA extracted from the organotin-treated Raji cells relative to the control.

***Studies on the DNA of Raji cells***

Upon electrophoresis using 0.8% agarose gel, a difference in the DNA pattern between the control and organotin-treated samples was observed: the DNA band of the control sample was located at around 23 kbp,

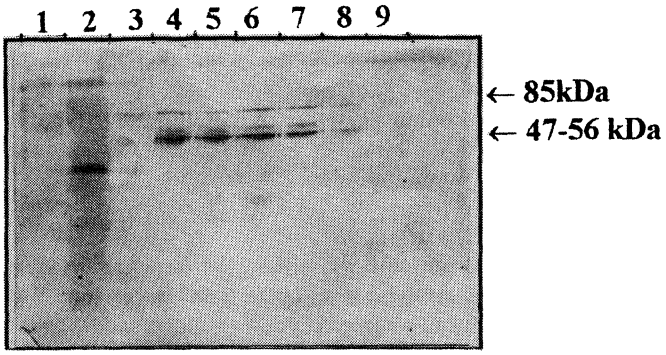

**Fig. 2: Western Blot depict of the suppression of EBV-EA(R) (85 kDa) and EA(D) (47-56 kDa) by [TPTCC•QuinO]**

***Loading sequence***: Lanes: (1) empty;(2) prestained marker-low range molecular weight proteins;(3) Control (untreated) Raji cells;(4) & (5) Control+TPA+sodium butyrate;(6) Raji cells+TPA+sodium butyrate+organotin 50 ng/mL ; (7) Raji cells+TPA+sodium butyrate+organotin 100 ng/mL ; (8) Raji cells+TPA+sodium butyrate+organotin 200ng/mL ; (9) Raji cells +TPA + sodium butyrate +organotin 400 ng/mL.

while for the case of the treated sample an immobile band, perhaps obstructed by the pore size of the gel, was seen at a higher molecular weight (*Fig. 3*). While this is not necessarily indicative of a chemical interaction between the organotin and the DNA, to enable a better appraisal of this possibility we subjected the extracted DNA to cleavage by restriction enzymes. The restriction enzymes, *Hind III* and *Eco R1*, which are known to

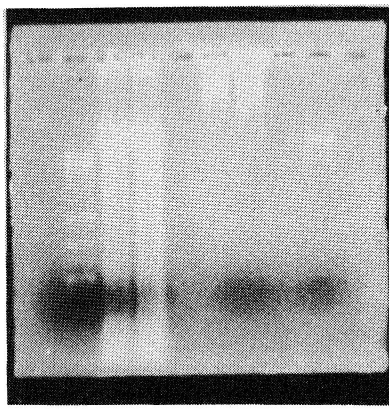

**Fig. 3: Agarose gel electrophoregram of extracted DNA from untreated (control) and organotin-treated Raji cells**

***Loading sequence***: Lanes: (1) empty; (2) 1 kb DNA ladder; (3) & (4) DNA from control sample; (5) empty; (6) & (7) DNA from organotin-treated sample; (8) λ *HIN*D *III* marker (0.12-23.1 kb)

have different specific recognition sites on DNA, were used for this purpose. In the case of the control sample, electrophoregram of the DNA excised by *Hind III* digestion showed a smear of several indistinguishable bands (10 kb and below) but only a single band at around 10 kb was noticed for the treated sample (*Fig. 4, left*). This suggests an inability of the restriction enzyme to cleave the apparently modified DNA in the latter case. A similar result (*Fig. 4, right*) was also obtained for the case of the *Eco R1* cleavage.

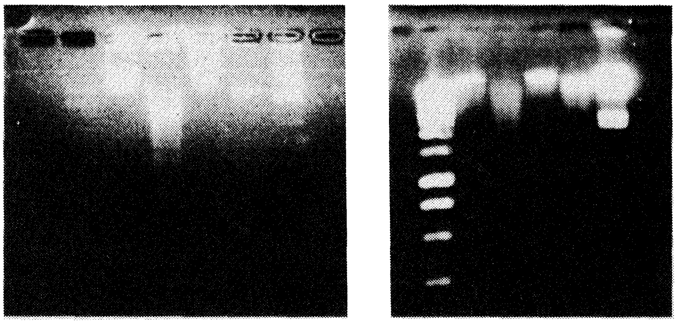

**Fig. 4: Agarose gel electrophoregrams of extracted DNA from control (incubation only with TPA- sodium butyrate) and treated (incubation with TPA-sodium butyrate and organotin) Raji cell samples following digestion with *HIN*D *III* (*left*) and *Eco R1* (*right*)**

***Loading sequence:*** Lanes: (1) empty; (2) 1 kb DNA ladder; (3) control sample undigested with restriction enzyme; (4) control sample digested with restriction enzyme; ( 5) treated sample undigested with restriction enzyme ; (6) treated sample digested with restriction enzyme ; (7) λ *HIN*D *III* marker

To rule out the possibility that the organotin might have itself partially fragmented the DNA and yielded a low molecular weight fraction that perhaps had escaped detection on agarose gel, a test was performed using a different DNA substrate, namely, the circular plasmid pBR322. This is a 4363 bp vector with unique recognition sites for *Hind III* cleavage in its $Tc^r$ (tetracycline resistant) gene. Incubation of the plasmid DNA with this restriction enzyme yielded a single band upon analysis by agarose gel electrophoresis, in contrast to two configurationally discernible DNA bands which were observed for each of the untreated plasmid and the organotin- incubated plasmid.

In the context of the above observations, it was of interest to determine whether the BMLF-1 gene specific to the EBV-EA protein had been rendered ineffective as a result of the treatment of the Raji cells with the organotin. Applying the polymerase chain reaction, this target gene was amplified in the control and

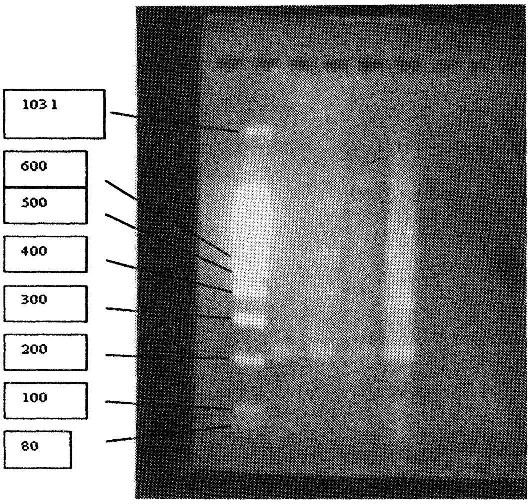

**Fig. 5: Agarose gel electrophoresis analysis of the PCR product from the amplification of the BMLF-1 gene performed on DNA extracted from organotin-treated and untreated (control) Raji cells**
***Loading sequence***: Lanes: (1) empty; (2) 100 bp DNA ladder; (3-6) control sample (previously incubated only with TPA-sodium butyrate); (7) treated sample (previously incubated with TPA-sodium butyrate and organotin (OT)) (1µg/mL OT); (8) treated sample (2 µg/mL OT); (9) treated sample ((3 µg/mL OT).

treated Raji samples using the primer set having the sequences shown below:

5 Oligo: 5' CTT GGA GAC AGG CTT AAC CAG ACT CA 3'

3 Oligo: 5' CCA TGG CTG CAC CGA TGA AAG TTA T 3'

The amplified samples were then subjected to electrophoresis in 1.5% agarose gel. As depicted in *Fig. 5*, the treated samples drew a blank in the electrophoregram relative to the control which yielded the characteristic 265 bp band of the BMLF-1 gene, suggesting that the organotin had affected the expression of this viral gene as a result of its punitive interaction with the Raji cell DNA. Although the evidence for the interaction of the organotin with DNA is only indirect, the result interestingly is at variance with previously reported observations of the selective accumulation of organotins in the Golgi apparatus and endoplasmic reticulum region of cells rather than in the nucleus[4].

**Acknowledgements**
Financial support from the National Science Council for Research and Development (Grant No.09-02-03-0142) is gratefully acknowledged. We thank the Institute of Biological Sciences for extending to us laboratory facilities in the pursuit of this work.

**References**

1. M.Gielen, P.Lelieveld, D.de Vos and R.Willem. In *"Metal Complexes in Cancer Chemotherapy"* (Ed: B.K.Keppler), VCH, Weinheim, 1993, Chapter 17, p.383; M.Gielen, *Coord.Chem.Rev.*, **151** (1966) 41.
2. M.Gielen, A.El Khloufi, M.Biesemans, A.Bouhdid, D.de Vos, B.Mahieu and R.Willem, *Metal-based Drugs*, **1** (1994) 305; M.Gielen, A.El Khloufi, M.Biesemans, F.Kayser and R.Willem, *Appl.Organomet.Chem.*, **7** (1993) 201, and references therein.
3. Y.Arakawa. In *"Chemistry and Technology of Silicon and Tin"* (Eds: V.G.Kumar Das, S.W.Ng and M.Gielen), Oxford Sc.Publ., Oxford, 1992, Chapter 23, p.319.
4. Y.Arakawa. In *"Chemistry of Tin"* (Ed: P.J.Smith), 2nd edn., Blackie Academic & Professional, London, 1998, Chapter 10, p.388.
5. A.J.Crowe, *Metal-based Drugs*, **1** (1989) 103.
6. S.W.Ng, V.G.Kumar Das and M.Gielen, *Appl.Organomet.Chem.*,**6** (1992) 489; S.W.Ng, V.G.Kumar Das, M.Gielen and E.R.T.Tiekink, *ibid*, **6** (1992) 19; S.W.Ng, V.G.Kumar Das, J.Holecek, A.Lyeka, M.Gielen and M.G.B.Drew, *ibid*, **11** (1997) 39.
7. M.J.Porter, J.K.Field, J.C.K.Lee, S.F.Leung, D.Lo and C.A.van Hasselt, *Anticancer Res.*, **14** (1994) 1357.

8. G.Niedobitek, M.L.Hansmann, H.Herbst, L.S.Young, D.Dieneman, T.Finn, C.A.Hartmann, S.Pitteroff, A.Welt, I.Anagnostopoulos, R.Friedrich, H.Lobeck, L.K.Sam, I.Aranjo, A.B.Rickinson and H.Stein, *J.Pathology*, **165** (1991) 17.
9. S.D.Kottaridis, E.Panatopoulou, I.Diamantis, I.Goula, J.Danilidis and G.Fountzilas, *Anticancer Res.*, **16** (1996) 785.
10. J.Luka, *J.Virological Methods,* **21** (1988) 223, and references therein.
11. Q.Liu and W.C.Summers, *J.Virology*, **63** (1989) 5062.
12. J.Countryman, H.Jenson, R.Seibl, H.Wolf and G.Miller, *J.Virology*, **61** (1987) 3672.
13. M.Kawanishi and Y.Ito, *Cancer Lett.,* **16** (1982) 19.
14. G.Henle, W.Henle and G.Klein, *Int.J.Cancer*, **8** (1971) 272.
15. S.W.Ng and V.G.Kumar Das, *Trends in Organomet.Chem.*, **2** (1997) 107; *Acta Cryst.,* **C53** (1997) 1034
16. D.J.Holme and H.Peck, *"Analytical Biochemistry"*, 2nd edn., 1993, Chapter 13, Longman Scientific & Technical, Harlow (UK)
17. I.Saito, Bo Servenius, T.Compton and R.I.Fox, *J.Exp.Med.*,**169** (1989) 2191
18. G.R.Pearson, B.Vroman, B.Chase, T.Sculley, M.Hummel and E.Kieff, *J.Virology*,**47** (1983) 193

**Received: October 31, 2000 - Accepted: November 28, 2000 - Received in revised camera-ready format: November 30, 2000**

